# A Triad of Highly Divergent Polymeric Immunoglobulin Receptor (*PIGR*) Haplotypes with Major Effect on IgA Concentration in Bovine Milk

**DOI:** 10.1371/journal.pone.0057219

**Published:** 2013-03-11

**Authors:** Sarah Berry, Wouter Coppieters, Stephen Davis, Alayna Burrett, Natalie Thomas, David Palmer, Van Kelly, Vladimir Obolonkin, Kathryn Sanders, Richard Spelman, Michel Georges, Klaus Lehnert, Russell Snell

**Affiliations:** 1 ViaLactia Biosciences, Auckland, New Zealand; 2 School of Biological Sciences, University of Auckland, Auckland, New Zealand; 3 Livestock Improvement Corporation, Hamilton, New Zealand; 4 Unit of Animal Genomics, GIGA-R & Faculty of Veterinary Medicine, University of Liège (B34), Liège, Belgium; Yale School of Public Health, United States of America

## Abstract

The aim of this study was to determine a genetic basis for IgA concentration in milk of *Bos taurus*. We used a Holstein-Friesian x Jersey F2 crossbred pedigree to undertake a genome-wide search for QTL influencing IgA concentration and yield in colostrum and milk. We identified a single genome-wide significant QTL on chromosome 16, maximising at 4.8 Mbp. The polymeric immunoglobulin receptor gene (PIGR) was within the confidence interval of the QTL. In addition, mRNA expression analysis revealed a liver *PIGR* expression QTL mapping to the same locus as the IgA quantitative trait locus. Sequencing and subsequent genotyping of the *PIGR* gene revealed three divergent haplotypes that explained the variance of both the IgA QTL and the *PIGR* expression QTL. Genetic selection based on these markers will facilitate the production of bovine herds producing milk with higher concentrations of IgA.

## Introduction

Transmission of maternal immunoglobulins to offspring via colostrum and milk influences neonatal health, providing essential protection against environmental pathogens [1]–[3]. Further, the improvement of human nutrition, including infant nutrition, is an important target for food producers. Although bovine milk is the predominant ingredient for most commercially prepared infant formulae, significant compositional differences between human and bovine milk exist. Accordingly, strategies to make the composition and function of infant formula more similar to that of human milk may be useful.

IgA is the most abundant immunoglobulin (Ig) in human colostrum and milk, comprising approximately 90% of the total Ig, whereas only 9% of the total Ig in bovine colostrum and milk is IgA [2]. The concentration of Ig in bovine milk and colostrum varies at different stages of lactation, with early colostrum secretions containing the highest concentrations [4]–[5]. Colostrum IgA concentrations follow this pattern with the maximum concentration reached during the early colostrum period [4]. Variation in IgA concentration within and between bovine breeds [6]–[8] has been observed, but the basis for this phenotypic variation has not been elucidated. More recently a possible genetic basis for natural antibody titres in bovine milk was also suggested [9].

Genetic diversity within an agricultural species may provide opportunity for improvements in production, disease resistance and product differentiation options. To explore this potential within dairy breeds, a Holstein-Friesian x Jersey crossbred trial was conducted, enabling the discovery of genes and mutations associated with variation in milk composition. We have previously shown that natural genetic variation can be successfully used to inform bovine breeding decisions [10]–[12]. One strategy to increase the concentration of IgA in bovine milk, to more closely match the composition of human milk, is the use of genetic selection, allowing the generation of specialised herds or dairy products.

Our hypothesis was that phenotypic variation in IgA content of bovine colostrum or milk would be, in part, genetically determined. Therefore, our aim was to identify loci associated with IgA, and to establish a selection tool that would allow colostrum and milk IgA to be increased. We searched for chromosomal regions associated with colostrum IgA content and identified a candidate gene, polymeric immunoglobulin receptor (PIGR). We demonstrate that polymorphisms within this gene explain more than 25% of the phenotypic variation for colostrum IgA and consequently provide the ability to increase milk IgA content through the use of marker-assisted selection.

## Materials and Methods

### Ethics Statement

Ethics approval for all sample collection procedures, manipulations and measurements performed on the animals was granted by the Ruakura Animal Ethics Committee, Hamilton, New Zealand. No animals were sacrificed during this study. The samples collected were milk and colostrum samples, which were collected by sub-sampling of milk and colostrum collected during industry-standard milk collections; blood samples, which were collected by venipuncture of the coccygeal vein, in accordance with procedures approved by the ethics committee, and needle biopsy samples of the fat and liver tissue, collected in accordance with procedures approved by the ethics committee. The farm was owned by our research group and managed by professional farm staff.

### Trial Pedigree design

A Holstein-Friesian x Jersey crossbred trial was conducted using an F2 pedigree design with a half-sibling family structure, as previously described [12]–[15]. Briefly, reciprocal crosses of Holstein-Friesian and Jersey animals were carried out to produce six F1 bulls of high genetic merit. Eight-hundred and sixty four F2 female progeny were then produced through mating of high genetic merit F1 cows with these F1 bulls. The herd was formed over two years, producing two cohorts, and a total of 724 F2 cows entered their second lactations. The animals were managed on a single farm under typical dairy farming practices in New Zealand using a seasonal, pasture-based system.

### Colostrum and milk sample collection

All measurements for the composition of colostrum and milk were taken during the cows' second lactation. Animals were milked twice daily, using standard dairy industry practice and the volume of milk was recorded at each milking using electronic milk meters. The animals were managed on a seasonal system, with lactation beginning after calving in spring. Colostrum samples were obtained at the second and the eighth milking after calving (days one and four of lactation, respectively) for analysis of IgA and IgG content. Milk samples were also obtained at mid-lactation (a fixed date approximately three months after calving) for analysis of IgA and IgM, as well as milk fat content and milk protein content, and somatic cell count (SCC). On each day that mid-lactation samples were collected, sub-samples from the morning and afternoon milk collections were combined to make a single composite milk sample for each animal. Total fat and total protein contents, and somatic cell count (SSC), a measure of mastitis, were assessed during standard herd testing procedures by Fourier transform infrared spectroscopy using a MilkoScan FT120 (Foss, 2006; Foss, Hillerød, Denmark). In addition, mid-lactation milk samples from a subset of F2 animals (n = 38) were retained for analysis of secretory component. Live-weight was obtained from each cow during the week of milk sampling.

### Analysis of immunoglobulins in milk

IgA concentration was measured using the bovine IgA ELISA quantitation kit (Bethyl Laboratories Inc, Montgomery, Texas, USA), as per manufacturer's instructions. A reference standard of bovine serum, containing 0.18 mg/mL IgA was used to calibrate the assay for the colostrum samples. IgM was measured using a bovine IgM ELISA quantitation kit (Bethyl Laboratories Inc, Montgomery, Texas, USA; catalogue number E10-101) as per manufacturer's instructions. A reference standard of bovine serum, containing 2.5 mg/mL IgM was used to calibrate the assay for the colostrum samples. IgG was measured using a nephlometric assay, as previously described [16]. IgA and IgG was measured in a total of 661 colostrum samples at both the second and eighth milkings. IgA and IgM were measured in 661 mid-lactation milk samples.

### Analysis of Secretory component in milk

Secretory component, the soluble fragment of *PIGR*, an IgA receptor, was measured in a subset (n = 38) of the 661 milk samples collected at mid-lactation. Whey was prepared by the addition of acetic acid to a final concentration of 1% followed by centrifugation at 3,000×g, 20 min, 8°C. The whey samples were reduced, alkylated with iodoacetamide, and digested with trypsin. The tryptic peptides of whey proteins and the synthetic peptide AAPAGAAIQSR, which corresponds to a tryptic peptide of secretory component, were analysed by reverse phase high performance liquid chromatography using an Agilent Technologies 1100 series capillary liquid chromatograph fitted with an Agilent Technologies Zorbax SB-C18 300 Å 0.3×150 mm 3.5 µm column coupled to an Agilent Technologies model SL ion trap mass spectrometer with an electrospray ionisation interface. The Zorbax column was developed with a gradient of 4% to 56% acetonitrile in 0.1% formic acid/0.005% heptafluorobutyric acid, over 27 minutes at a flow rate of 4 µl/min. The mass spectrometer was operated in single ion monitoring mode which was set to isolate and fragment the doubly protonated ion of peptide AAPAGAAISQR observed at 506.8 m/z; fragment ions were observed by scanning the mass range from 430 m/z to 810 m/z. The predominant fragment ion observed at 435.9 m/z, corresponding to the doubly protonated y9 ion, was used for quantitation; ions observed at 623.3 m/z, corresponding to the b8 ion, and at 704.2 m/z, corresponding to the y7 ion, were used as qualifiers. The peptide AAPAGAAIQSR observed in samples of trypsin-digested whey was quantitated by comparison to a standard curve constructed using the synthetic peptide AAPAGAAIQSR.

### Liver biopsy, mRNA preparation and microarray analysis

Liver and adipose tissue biopsies were taken from a subset of the F2 Holstein-Friesian x Jersey crossbred cows. Approximately 480 cows were biopsied, during the third lactation, ten weeks after parturition. Biopsy procedures were in accordance with procedures approved by the Ruakura Animal Ethics committee and were conducted as previously described [17]. Biopsy tissue (approximately 0.5 cm^3^) was placed in a container with 10 volumes RNAlater (Ambion), stored overnight at 4°, and then frozen at −20°. After thawing, the tissue was removed from the RNAlater and homogenised in 750 µl TRIzol (Life Technologies) for liver samples, and 750 µl QIAzol (Qiagen) for adipose samples. RNA was then extracted according to manufacturer's instructions. The RNA was quantified using a NanoDrop (ThermoScientific), and a quality assessment was made using an RNA 6000 BioAnalyzer chip (Agilent). Between 5 and 8 µg total RNA was labelled using the Affymetrix One-Cycle Target Labeling and Control Reagents Kit (Affymetrix, P/N 900493). 10 µg of labeled, fragmented cRNA was hybridized overnight to a GeneChip Bovine Genome Array (Bovine 3′ arrays, Affymetrix), and the array was processed and scanned according to the manufacturer's instructions. The microarray contained 24,027 probe sets representing more than 23,000 transcripts and includes approximately 19,000 UniGene clusters. There were 11 probe pairs per probe set.

Following microarray hybridisation and scanning, gene expression data files (CEL files) were obtained for analysis. The R (version 2.13.1, [18]) computational environment and “affy” library were used as recommended by Affymetrix to assess data quality. Of the 855 data files, 26 were found to have RNA degradation and were consequently removed from further analysis. The remaining 829 data files (359 for adipose tissue, 470 for liver tissue, 334 animals with both liver and fat observations) were processed as one batch using the Affymetrix Robust Multi-Array Average (RMA) normalisation procedure. The output was then adjusted using REML, separated by tissue type and fitted for biopsy date and RNA preparation date as fixed effects. In addition, sire was fitted as a random effect. The resulting residuals were used for QTL identification as described below. Data are presented as adjusted means ± standard error.

### Genotyping

Genomic DNA was prepared from whole blood, 1661 animals within the trial pedigree, including 846 F2 daughters, the six F1 sires, 796 F1 dams, and 13 selected F0 sires. The entire pedigree was genotyped using the Illumina BovineSNP50 BeadChip (Illumina, San Diego, USA) which assayed 54,000 SNPs. Additional genotyping of SNPs discovered within the *PIGR* gene was performed, using the single base extend 1 method and iPlex gold chemistry (Sequenom).

### Genome sequencing of F1 sires

Genomic DNA for whole genome sequencing was prepared from whole blood of the six F1 sires, using a phenol-chloroform extraction. Sample preparation and whole genome sequencing was carried out by Illumina FastTrack, using the HiSeq 2000 system. A total of 958Gbase of data (100 bp Paired End, ∼320 bp insert size, V2 chemistry) was obtained (average 159.7 Gbase per F1 sire).

Reads were aligned to the Bos Taurus UMD3.1 (bosTau6) genome sequence with BWA version 5 [19] using the default parameters. Indel realignment, duplicate removal and SNP/INDEL detection was performed on the six alignments simultaneously using the Genome Analysis Toolkit (GATK; [20]). GATK was also used to recalibrate the quality scores of the variants [21]. Variants with a VSQLOD below one were not analysed.

### QTL mapping

QTL mapping was conducted using a previously described mixed model that includes a locus-specific haplotype effect (random) as well as an individual polygenic effect (random) to correct for stratification [22]. Genotypes were phased using both familial (Mendelian segregation and linkage) and population (linkage disequilibrium (LD)) information and assigned to 20 ancestral haplotypes using a Hidden Markov Model [22]. The haplotype effect in the mixed model corresponded to the effect of the 20 ancestral haplotypes, which were considered uncorrelated. The covariances between the individual polygenic effects corresponded to twice the coefficient of kinship estimated from pedigree records. Variance components were estimated using AIREML [23]. The statistical significance of the haplotype effect was estimated using a likelihood ratio test (LRT = 2LN(LR)) comparing the likelihood of the data under the full model with that under a model without haplotype effect. The LRT was assumed to be distributed under the null hypothesis as a mixture of chi-squared with 1 and 2 degrees of freedom [24]–[25]. Consequently, the threshold for genome-wide significance (expected to be exceeded by chance once per 20 genome scans) was set at 24, corresponding to a Bonferroni-corrected p-value of 9.6×10^−7^≈0.05/54,000, while the genome-wide suggestive threshold (expected to be exceeded by chance on average once per genome scan) was set at 19.8, corresponding to a Bonferroni-corrected p-value of 8.5×10^−6^≈0.37/54,000.

### Statistical analysis of milk phenotypes

Data analysis was performed using R (version 2.13.1, [18]). The final dataset included observations for 661 animals for all phenotypes except secretory component, for which we had observations in 38 animals. The phenotypic variation of IgA concentration, milk volume and IgA yield (IgA concentration multiplied by milk volume) was described using analysis of variance, where milking number (second or eighth), sire (sires 1–6), calving date and cohort (cohort one or two, determined by birth year) were fitted as fixed effects. The effect of *PIGR* “genotype of haplotype” (I/I, I/II, I/III, II/II, II/III, III/III) on colostrum IgA and IgG, measured at the 2nd and 8th milkings, and on IgA, IgM, secretory component, milk fat content, milk protein content, milk volume and somatic cell count, all measured at mid-lactation, was determined using a generalized linear model. *PIGR* “genotype of haplotype”, sire and cohort were fitted as fixed effects, while calving date was fitted as a continuous covariate. A significance level of p = 0.05 was used throughout, and LS means and their corresponding standard errors were evaluated using the Effects package in R (version 2.13.1, [18]).

## Results

### Phenotypic variation of colostrum IgA

The average IgA concentration in colostrum at the second milking was 1.65±0.04 mg/mL and, as expected, decreased significantly by the eighth milking to 0.43±0.04 mg/mL (P = 1.54×10^−75^). Milk volume at the 2nd milking was 8.07±0.10 L and at the 8th milking was 9.82±0.10 L (P = 3.76×10^−31^). The yield, or total amount, of IgA produced also differed significantly between the second and eighth milkings (12.85±0.37 mg/milking vs. 4.10±0.37 mg/milking, P = 5.35×10^−58^). The concentration of IgA in colostrum differed significantly according to transmitting sire, at both the second and the eighth milkings (P = 7.96×10^−6^ and P = 7.52×10^−9^ respectively), as did the yield of IgA at both the second and eighth milkings (P = 1.38×10^−5^ and 1.18×10^−6^ respectively). IgA concentration in colostrum was also significantly influenced by cohort (0.38±0.01 mg/mL vs. 0.46±0.01 mg/mL, P = 0.013) and by calving date (P = 0.0063, data not shown). IgA yield in colostrum was not significantly influenced by cohort (P = 0.59, data not shown) or by calving date (P = 0.559, data not shown). The average of IgA concentration in mature milk, measured at mid-lactation, was 0.69±0.03 mg/mL. Milk volume at mid-lactation was 16.76±2.80 L, and thus the yield per day of IgA at this time-point was 11.35±0.21 mg. The concentration and yield of IgA at mid-lactation differed significantly according to transmitting sire (P = 0.005 and 5.68×10^−7^ respectively). IgA concentration at mid-lactation was also significantly influenced by cohort (0.61±0.012 mg/mL vs. 0.75±0.014 mg/mL, P = 3.10×10^−7^) and by calving date (P = 1.01×10^−5^; data not shown). IgA yield at mid-lactation was significantly influenced by cohort (10.18±0.17 mg/milking vs. 12.3±0.23 mg/milking, P = 2.36×10^−8^) but not calving date (P = 0.663, data not shown).

### A QTL with major effect on colostrum and milk IgA maps to bovine chromosome 16

**Figure 1:**
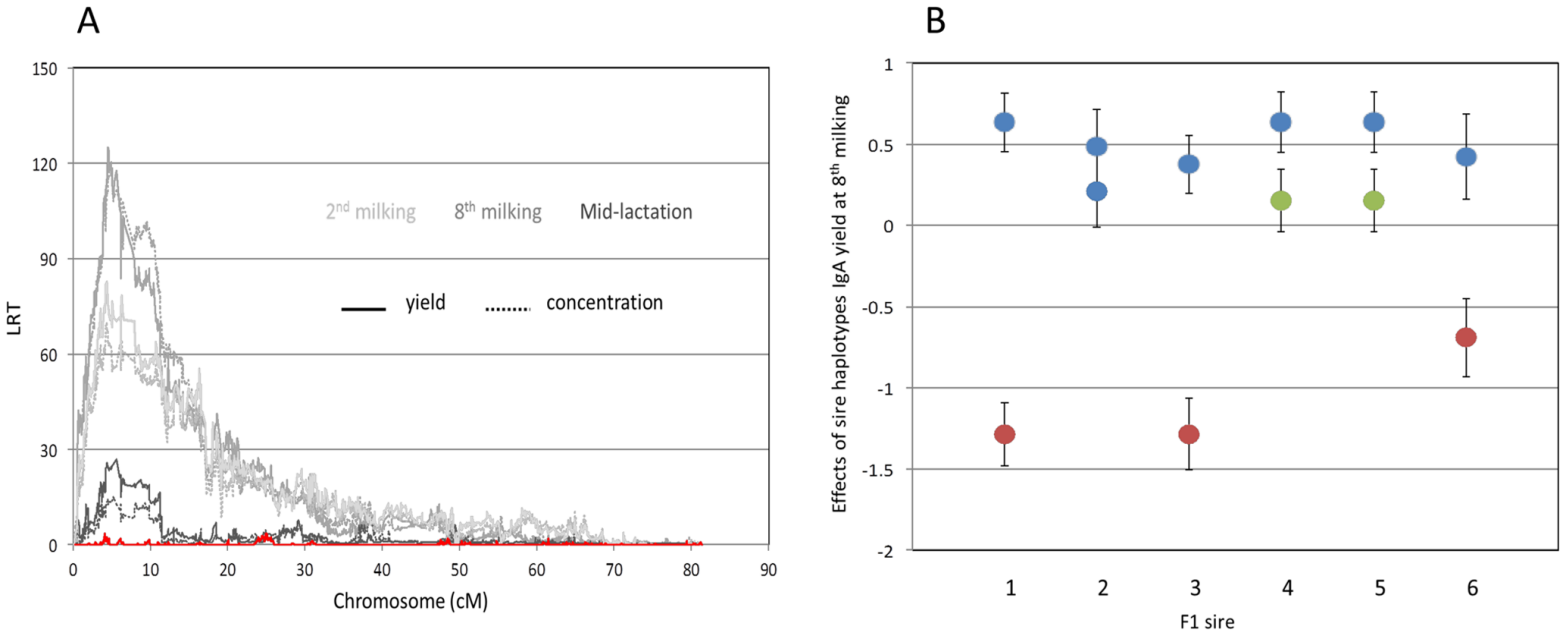
Chromosome 16 QTL for colostrum and milk IgA content. (**A**) Location scores (LRT = likelihood ratio test) obtained when scanning bovine chromosome 16 for QTL influencing IgA concentration (dotted lines) and yield (continuous line) in colostrum collected at the 2^nd^ milking (“2^nd^ colostrum”; light gray), colostrum collected at the 8^th^ milking (“8^th^ colostrum”; dark gray) and mid-lactation milk (black) using a mixed-model based approach that simultaneously extracts linkage and LD information. The red curve corresponds to the location scores obtained for IgA yield of 8^th^ colostrum (giving the strongest signal in single QTL analysis) when adding the effect of *PIGR* haplotype (I, II and III; cfr. Fig. 4) in the model. (**B**) Effect (± SEM) of the haplotypes of the six F1 sires on IgA yield of the 8^th^ colostrum (mg/milking). The haplotypes are labeled according to their corresponding *PIGR* genotype (I: blue; II: green; III: red; cfr. Fig. 4).

**Figure 2:**
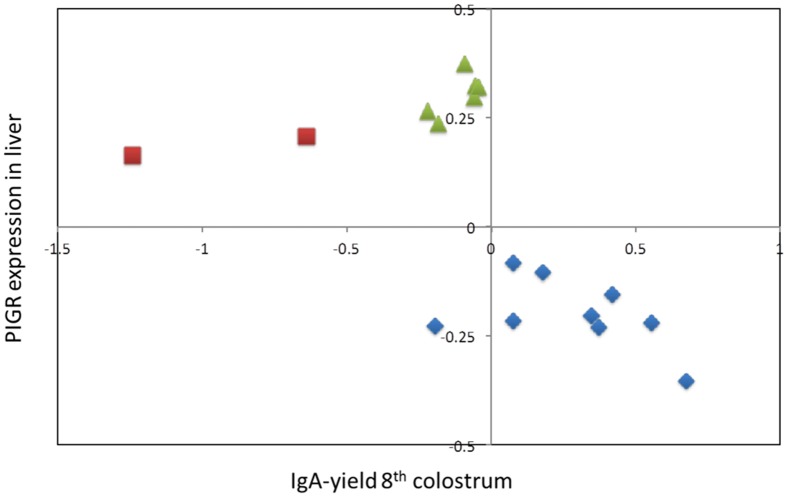
Effects of hidden haplotype states. Bivariate effects (Y-axis: *PIGR* expression in liver; X-axis: IgA yield of 8^th^ colostrum) of the 20 Hidden Haplotype States at the most likely position of the 8^th^ colostrum IgA yield QTL (4,533,875). Hidden Haplotype States are labelled according to their *PIGR* haplotype (I: blue; II: green; III: red; cfr. Fig. 4).

We scanned the genome for QTL influencing IgA concentration and yield in colostrum and milk using a previously described haplotype-based method that simultaneously extracts linkage and LD information and accounts for population stratification [22]. The phenotypes used for this QTL mapping are in Table S1 and genotypes are in Table S2, with marker positions in Table S3. This analysis revealed a single genome-wide significant QTL with major effect on IgA concentration and yield in both colostrum and milk on chromosome 16 (Fig. 1A and Fig. S1). The most likely position of the QTL ranged from position 4,305,200 to 5,540,018 (UMD3.1/bosTau6) depending on the phenotype considered. The 95% confidence interval for the QTL, defined with the LRT-4.6 ( = lod-2) drop-off method applied to the phenotype that yielded the highest LRT, (colostrum IgA yield at the eighth milking; LRT = 124.9 at chromosome position 4,533,875); spanned ∼162 Kb (chromosome position 4,466,778–4,628,745). The effects of hidden haplotype state on colostrum IgA yield at the 8th milking ranged from −1.24±0.037 to +0.68±0.077 mg (Fig. 2). The substitution effects of the haplotypes carried by F1 sires 1, 3 and 6 were large (between 1.19 and 1.67 mg), by F1 sires 4 and 5 intermediate (0.45 mg), and by F1 sire 2 virtually zero (Fig. 1B).

In addition to this significant QTL on chromosome 16, we obtained tentative evidence for a QTL influencing IgA yield at the 2^nd^ colostrum collection on chromosome 4 (Fig. S1).

### An expression QTL (eQTL) influencing hepatic *PIGR* expression coincides with the IgA QTL

**Figure 3:**
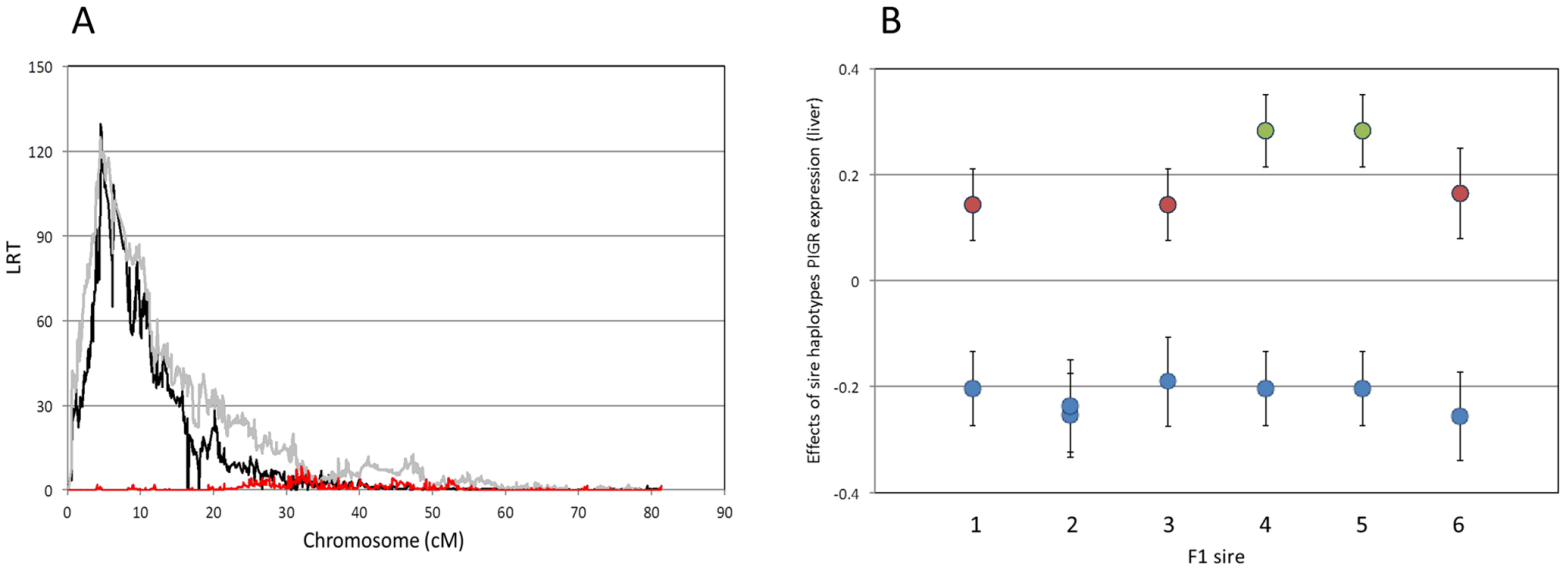
Chromosome 16 eQTL for *PIGR* mRNA expression. (**A**) Location scores (LRT = likelihood ratio test) obtained when scanning bovine chromosome 16 for QTL influencing *PIGR* transcript levels (black line) in liver using a mixed-model based approach that simultaneously extracts linkage and LD information. The red curve corresponds to the location scores obtained for *PIGR* mRNA expression level (liver) when adding the effect of *PIGR* haplotype (I, II and III; cfr. Fig. 4) in the model. The gray line corresponds to the location score obtained for IgA yield of the 8^th^ colostrum (cfr. Fig. 1A). (**B**) Effect (± SEM) of the haplotypes of the six F1 sires on *PIGR* expression level in liver. The haplotypes are labeled according to their corresponding *PIGR* genotype (I: blue; II: green; III: red; cfr. Fig. 4).

The 95% CI for the IgA QTL encompasses seven annotated genes: *IL19, IL20, ITM2C, PIGR, FCAMR, FAIM3* and *IL24*. *PIGR* encodes the polymeric immunoglobulin receptor, which has been previously implicated in the binding, and basal to apical translocation of IgA molecules in a variety of cell types including epithelial cells [26]–[27]. Thus *PIGR* was an excellent positional candidate gene. To examine whether the *PIGR* gene might be involved in determining the IgA QTL, we first took advantage of microarray data, available for adipose and hepatic tissue of 359 and 429 F2 cows, respectively. We analyzed the *PIGR* expression data using the same haplotype-based mixed model approach that was used for IgA, to search for QTL influencing *PIGR* expression. The phenotypes used for this QTL mapping are in Table S1 and genotypes are in Table S2, with marker positions in Table S3. We obtained a highly significant *cis*-eQTL (LRT = 145.24) at position 4,540,424, i.e. within the 95% confidence interval of the IgA QTL (eighth colostrum) and within the body of the *PIGR* gene (Fig. 3A). The effects of hidden haplotype state on *PIGR* expression ranged from −0.35±0.01 to +0.37±0.02 (arbitrary units; Fig. 2), and were very significantly correlated (p = 0.01) with their effect on IgA in colostrum and milk (Fig. 2). However, an increase in hepatic *PIGR* expression was associated with a decrease in IgA secretion into milk. Moreover, despite the correlation between the haplotype effects on *PIGR* expression and IgA, the substitution effects on *PIGR* expression of the haplotypes of the F1 sires did not match those on IgA amounts: they were large for sires 4 and 5 (0.48 arbitrary units), intermediate for sires 1, 3 and 6 (0.3187, 0.368 and 0.428 arbitrary units, respectively) and virtually zero for sire 2 (Fig. 3B).

### Genome sequencing reveals the segregation of three, highly divergent *PIGR* haplotypes

We took advantage of genome-wide resequencing data recently generated for the six F1 sires that will be described in more detail elsewhere. We focused our attention on ∼24 Kb spanning the 11 exons of the *PIGR* gene including ∼10 Kb of upstream and ∼3 Kb of downstream sequence. Sequence depth across this interval averaged 262 (range: 9–451). We analyzed the sequence data using GATK [20]–[21] and identified 269 sequence variants (251 SNP, 10 deletions, 8 insertions) within this interval. Of these, 258 (i.e. 98%) conformed to one of three segregation patterns in the F1 sires: (i) sires 1, 3 and 6 heterozygous (126 variants), (ii) sires 4 and 5 heterozygous (95 variants), and (iii) sires 1, 3, 4, 5 and 6 heterozygous (37 variants). The most parsimonious explanation of this finding is the segregation of three haplotypes (I, II, III) for which the F1 sires would have genotype I/II (sire 1), I/I (sire 2), I/II (sire 3), I/III (sire 4), I/III (sire 5) and I/II (sire 6) (Table S4). We developed genotyping assays for eight SNPs. Four of those (E3_903 at position 4543550, E4_3938 at position 4540515, E4_4029 at position 4540424, and E5_5183 at position 4539270) would have segregation pattern (i), two (3′UTR_9519 at position 4534484 and 3′UTR_9640 at position 4534363) segregation pattern (ii), and two (5′UTR_-33 at position 4544485 and 5′UTR_-9 at position 4544461) segregation pattern (iii). We genotyped the entire F2 pedigree and phased the resulting genotypes using Phasebook [22]. The three haplotypes predicted from the sequence data of the F1 sires indeed accounted for 98.2% of the chromosomes in the F2 generation (I: 50.3%; II: 12.4%; III: 35.5%).

**Figure 4:**
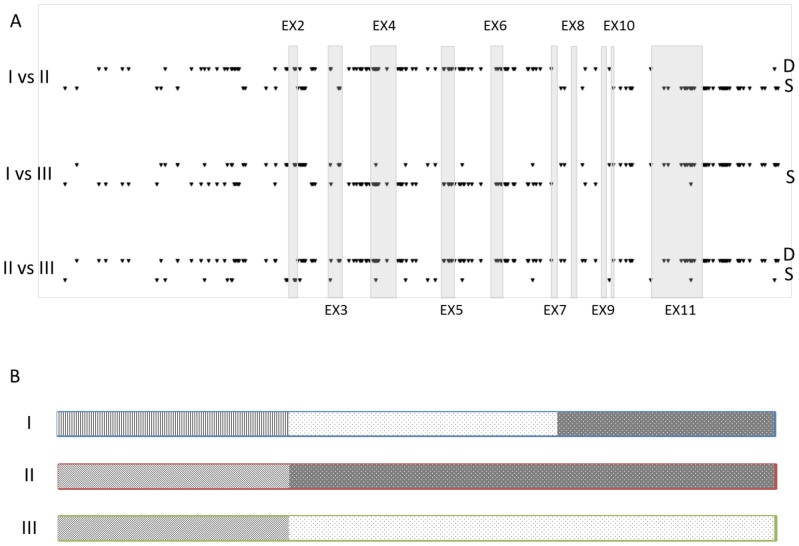
Sequence comparison of *PIGR* haplotypes. (**A**) Variant positions at which the corresponding pair of *PIGR* haplotypes ***D***iffer (upper line; “D”), or are the ***S***ame (lower line; “S”). The positions of the *PIGR* exons are marked by the transparent gray boxes. (B) Schematic representation of the three major *PIGR* haplotypes with indication of the positions at which they differ or not. Within the body of the *PIGR* gene, haplotype I is appears as a recombinant between haplotype II and III, which differ on average every 109 nucleotides. Upstream of the gene, the three haplotypes differ on average every ∼200 nculeotides.

Closer examination of the sires' sequences indicated that haplotypes II and III differ at 224/269 variant positions, corresponding to a remarkably high average nucleotide diversity (π) of 1/109. The differences between these two highly divergent haplotypes include 32 exonic variants of which 19 are non-synonymous (Table S4). Moreover, haplotype I appeared recombinant between these two highly divergent haplotypes: it is closely related to haplotype II at the 3′ end (downstream of exon 6; π = 1/705), while being closely related to haplotype III in the center (exon 4 – intron 6; π = 1/609). The relationship between the three haplotypes was less contrasted at the 5′ end of the *PIGR* gene (upstream of intron 3), the nucleotide diversity being ∼1/200 for all three haplotype comparisons (Fig. 4).

### The *PIGR* haplotype triad fully accounts for the IgA QTL and *PIGR* eQTL

**Figure 5:**
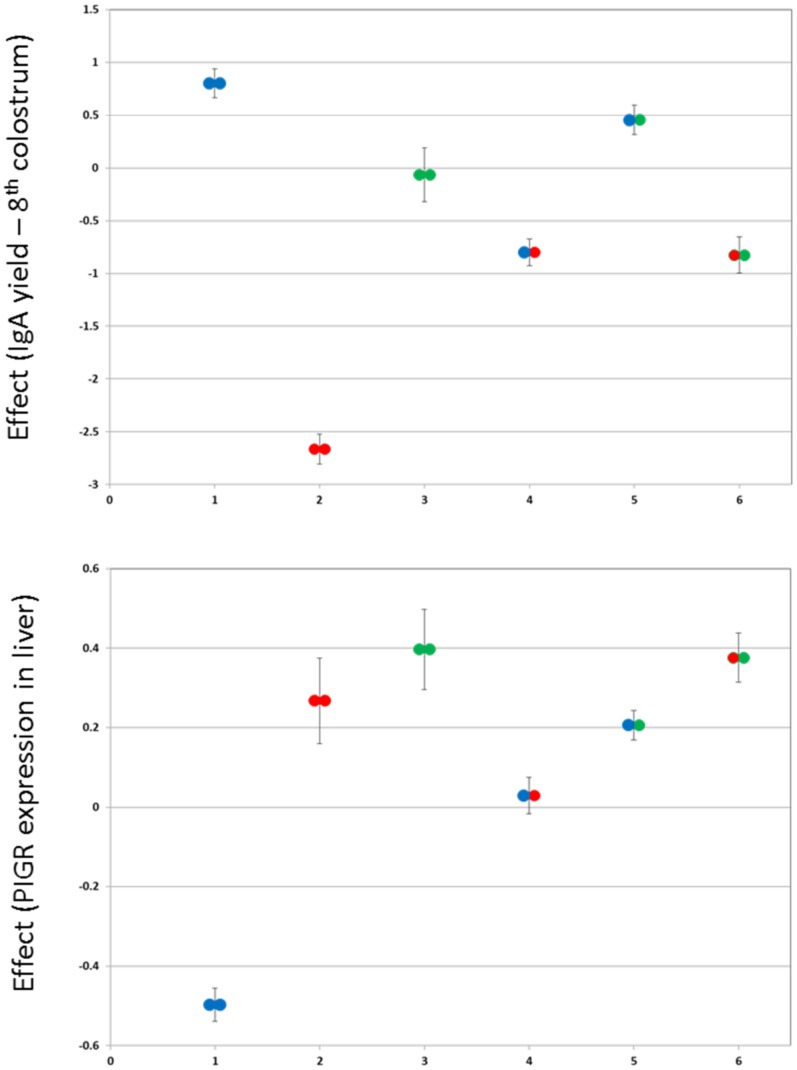
Effect of *PIGR* genotype on IgA yield and *PIGR* expression. Phenotypic effects (± SEM) of the six possible *PIGR* genotypes (I/I, II/II, III/III, I/II, I/III, II/III – I: blue; II: green; III: red; cfr. Fig. 4) on IgA yield of the 8^th^ colostrum (**A**) and *PIGR* expression in liver (**B**). In general the phenotypic mean of the heterozygotes is intermediate between the corresponding alternate homozygotes, supporting additivity.

We determined the correspondence between the hidden haplotype states used for the QTL analysis and the *PIGR* haplotypes. As can be seen from Fig. 2, the three *PIGR* haplotypes define three clusters of hidden states that are non-overlapping with respect to their bivariate effect on milk IgA and hepatic *PIGR* expression levels. When adding *PIGR* haplotype as a random effect in the QTL analyses, hidden haplotype effects on IgA amount and *PIGR* expression disappeared, indicating that *PIGR* haplotypes fully explain the identified QTL (Fig. 1A & 3A). *PIGR* genotype (I/I, I/II, I/III, II/II, II/III) accounted for ∼4% of the variance of IgA yield/concentration in the 2^nd^ colostrum milking, ∼20% in the 8^th^ colostrum milking, ∼17% in mid-lactation, while accounting for ∼35% of the variance in *PIGR* expression level in liver. *PIGR* haplotype effects on both IgA yield/concentration as well as on *PIGR* transcript levels appeared to largely act additively (intermediate phenotype of heterozygotes when compared to alternate homozygotes; Fig. 5).

### Effect of *PIGR* genotype on milk composition

We considered the effect of “genotype of haplotype” on milk composition. The concentration and yield of IgA in colostrum (at the second and eighth milkings), and in mature milk (mid-lactation) was significantly affected by genotype (Table 1). In particular, animals homozygous for haplotype II produced colostrum and milk with approximately one third the concentration of IgA than animals homozygous for haplotype I (Table 1). There was also a significant effect of haplotype on milk IgM concentration, with animals homozygous for haplotype II producing milk with approximately half the concentration of IgM than animals homozygous for haplotype I (Table 1). In contrast, there was no effect of *PIGR* haplotype on colostrum IgG concentration. No significant effect of genotype was observed for the amount of fat or protein in milk, daily milk yield, or somatic cell count (Table 1).

**Table 1:** Effect of *PIGR* haplotype on milk composition traits.

		Genotype of Haplotype						
	Phenotype	I/I	I/II	I/III	II/II	II/III	III/III	P-Value
**Colostrum, 2^nd^ milking**	IgA (mg/mL)	1.91 (0.12)	1.41 (0.15)	1.79 (0.12)	0.62 (0.33)	1.48 (0.18)	1.36 (0.29)	0.0013
	IgG (mg/mL)	7.5 (0.46)	8.82 (0.58)	8.31 (0.46)	9.25 (1.31)	9.24 (0.72)	7.28 (1.17)	0.2672
	Milk Volume (L)	8.33 (0.2)	7.97 (0.26)	8.15 (0.2)	7.84 (0.58)	7.88 (0.31)	7.26 (0.51)	0.4208
	IgA Yield (mg)	15.67 (0.95)	10.56 (1.2)	14.06 (0.96)	4.48 (2.72)	10.41 (1.48)	10.09 (2.43)	0.0001
**Colostrum, 8^th^ milking**	IgA (mg/mL)	0.51 (0.012)	0.36 (0.015)	0.47 (0.012)	0.16 (0.034)	0.34 (0.018)	0.37 (0.03)	4.51×10^−28^
	IgG (mg/mL)	1.04 (0.04)	1.19 (0.05)	1.13 (0.04)	1 (0.11)	1.2 (0.06)	1.01 (0.1)	0.0552
	Milk Volume (L)	10.06 (0.2)	9.4 (0.25)	9.86 (0.2)	9.88 (0.56)	9.62 (0.31)	10.49 (0.5)	0.2787
	IgA Yield (mg)	5 (0.13)	3.27 (0.16)	4.49 (0.13)	1.58 (0.36)	3.24 (0.2)	3.87 (0.32)	1.63×10^−26^
**Mid-Lactation**	IgA (mg/mL)	0.82 (0.024)	0.55 (0.03)	0.76 (0.024)	0.32 (0.068)	0.57 (0.037)	0.65 (0.061)	3.99×10^−17^
	IgM (mg/mL)	0.14 (0.005)	0.13 (0.006)	0.14 (0.01)	0.09 (0.01)	0.13 (0.01)	0.13 (0.01)	0.0099
	Secretory component^a^ (mg/mL)	9.69 (1.77)	3.51 (1.64)	5.62 (0.76)	3.79 (1.16)	5.73 (1.22)	N/A	0.1323
	Protein (%)	3.78 (0.02)	3.82 (0.02)	3.78 (0.02)	3.78 (0.05)	3.83 (0.03)	3.86 (0.04)	0.2571
	Fat (%)	5.29 (0.04)	5.29 (0.05)	5.31 (0.04)	5.32 (0.11)	5.29 (0.06)	5.55 (0.1)	0.2493
	Milk volume (L)	17.1 (0.18)	16.7 (0.23)	16.8 (0.18)	17.3 (0.51)	16.2 (0.28)	16.2 (0.46)	0.1036
	Somatic cell count (×1000)	148 (27)	121 (34)	111 (27.2)	93 (76.9)	131 (42)	107 (68.7)	0.9359

a n = 38 observations.

mean presented with standard error in brackets.

## Discussion

We have demonstrated that the *PIGR* gene is characterized by three common haplotypes that segregate at intermediate frequencies in both HF and Jersey, and that these fully account for (i) a QTL with major effect in milk IgA concentrations, and (ii) a *cis*-acting eQTL on *PIGR* transcript levels in adult liver. The IgA QTL is primarily due to the negative effect on milk IgA levels of haplotype II when compared to haplotypes I and III. The most striking difference between haplotype II, and haplotypes I and III, is a 7.3 Kb segment between exons 3 and 10, characterized by 85 genetic variants differentiating II versus I/III. These include 13 non-synonymous substitutions (Table S1). A likely hypothesis is that one or several of the structural variants in this segment directly affect the functionality of *PIGR* and cause the observed QTL effect. All of these variants are in near perfect LD in the analyzed sample. It is therefore impossible to genetically identify the causative variant(s) by association analysis. Further dissection of the molecular mechanisms underlying the corresponding effect will require the analysis of other populations and/or functional assays.

The *PIGR* eQTL effect is primarily due to the negative effect on hepatic transcript levels of haplotype I, when compared to haplotypes II and III. There are 43 variant positions for which haplotypes II and III are identical yet differ from haplotype I. A plausible hypothesis is that one or several of these (or variants in high LD with them yet outside of the considered region) are directly responsible for the eQTL effect observed in liver. It is noteworthy that five of the corresponding variants cluster within 300 bp from the presumed transcriptional start site, including one with Phastcons score of 0.5. Alternatively, the higher expression levels of haplotypes II and III (when compared to I) could depend on distinct regulatory variants.

In addition to these two major (e)QTL effects, our results support distinct minor effects, underlying (i) the differences between haplotypes I and III with regards to IgA concentrations, and (ii) the differences between haplotypes II and III with regards to *PIGR* expression. Haplotypes I and III differ most strikingly in the terminal 5.6 Kb of the *PIGR* gene, with 73 variant positions. However, none of these alters the *PIGR* open reading frame. Haplotype III shares one non-synonymous substitution differentiating it from haplotype I in common with haplotype II (*K167R*), and this could underlie the observed minor effect on IgA concentrations. As mentioned before, haplotypes II and III differ at a minimum of 224 positions. Several of these could contribute to the minor II to III eQTL effect. Of note, haplotypes II and III differ for three SNPs with Phastcons scores>0.98, ∼1.4 Kb upstream of the transcriptional start site.

Our data does not allow us to draw definitive conclusions with regards to the contribution of regulatory variants to the IgA QTL. The eQTL effect observed in liver may be tissue-specific and not accurately reflect relative *PIGR* expression in the mammary gland. However, the fact that (i) some haplotype contrasts are strong for IgA concentrations yet weak for expression (I versus III), and vice versa (II versus III), and (ii) increased *PIGR* expression is associated with decreased IgA concentrations, does not support a direct functional link between IgA QTL and *PIGR* eQTL.

The nucleotide diversity observed between *PIGR* haplotypes II and III (π = 1/109) is high. This divergence level is typically expected for haplotypes sampled in distinct sub-species rather than in the same species. A plausible hypothesis is that the two haplotypes originate from distinct bovine sub-species that have been independently domesticated and have subsequently undergone hybridization. This situation is reminiscent of the highly divergent *IGF2* haplotypes that have been observed in commercial European pig populations shown to respectively trace back to Asian and European wild boar populations [28].

It has been proposed that *PIGR* is also a transporter of IgM [27], [29]–[30]. In agreement with this observation, our data show a significant effect of the *PIGR* gene on the concentration of IgM in milk, suggesting that this receptor indeed is important for the secretion of IgM into bovine milk and that similar genetic mechanisms underlie phenotypic variation in both IgA and IgM. There was no effect of the *PIGR* gene on the concentration of IgG in milk.

Secretory IgA provides the first line of defence against environmental pathogens. Within the mammary gland, secretory IgA may play a dual role, providing protection against environmental pathogens causing mastitis [31]–[32] as well as providing critical immunity to the neonate. Therefore, it is interesting to speculate whether polymorphisms in the *PIGR* gene may also relate to disease resistance in the cow and the calf.

In conclusion, we describe mutations within the bovine *PIGR* gene which form a genetic basis for variation in the secretion of IgA into bovine colostrum and milk. Genetic selection based on these markers will facilitate the production of bovine herds producing milk with higher concentrations of IgA.

## Supporting Information

Figure S1 Genome-wide scan for QTL affecting IgA, IgG and IgM. Manhattan plots obtained using a haplotype-based mixed model that simultaneously extracts linkage and LD information and corrects for stratification. For antibody (IgA, IgG or IgM as indicated) concentration (left column) and yield (right column) in 2^nd^ colostrum, 8^th^ colostrum or mid-lactation milk. The LRT threshold for genome-wide significance is 24, while the genome-wide suggestive threshold is 20.
(TIF)

Table S1 Phenotype data used for QTL mapping. ID = unique animal identification number. Cohort = cohort 1 or 2, based on year of birth. Phenotypes listed are milk volume, IgA concentration, IgA yield, IgG concentration, and IgG yield for the second and eighth milkings, IgA concentration and yield at mid-lactation, PIGR mRNA expression in liver and fat tissue.
(XLS)

Table S2 Genotypes in the PIGR region. ID = unique animal ID. Genotypes are listed as 1 or 2 for both the paternal and maternal alleles.
(XLS)

Table S3 Marker map information. For the markers listed in Table S2, the bovine chromosome 16 map position (UMD3.1) is given, along with RS# if available.
(XLS)

Table S4 Sequence variant data for the six F1 sires. Sires are numbered 1001–1006. Sequence variant information is given in terms of reference allele and alternative allele, as well as reference amino acid and alternative amino acid where applicable.
(XLS)
