# Supplementary figures and images for: A Triad of Highly Divergent Polymeric Immunoglobulin Receptor (PIGR) Haplotypes with Major Effect on IgA Concentration in Bovine Milk
Source: PLoS One. 2013 Mar 11;8(3):e57219. doi: 10.1371/journal.pone.0057219 (PMC3594236; doi:10.1371/journal.pone.0057219)

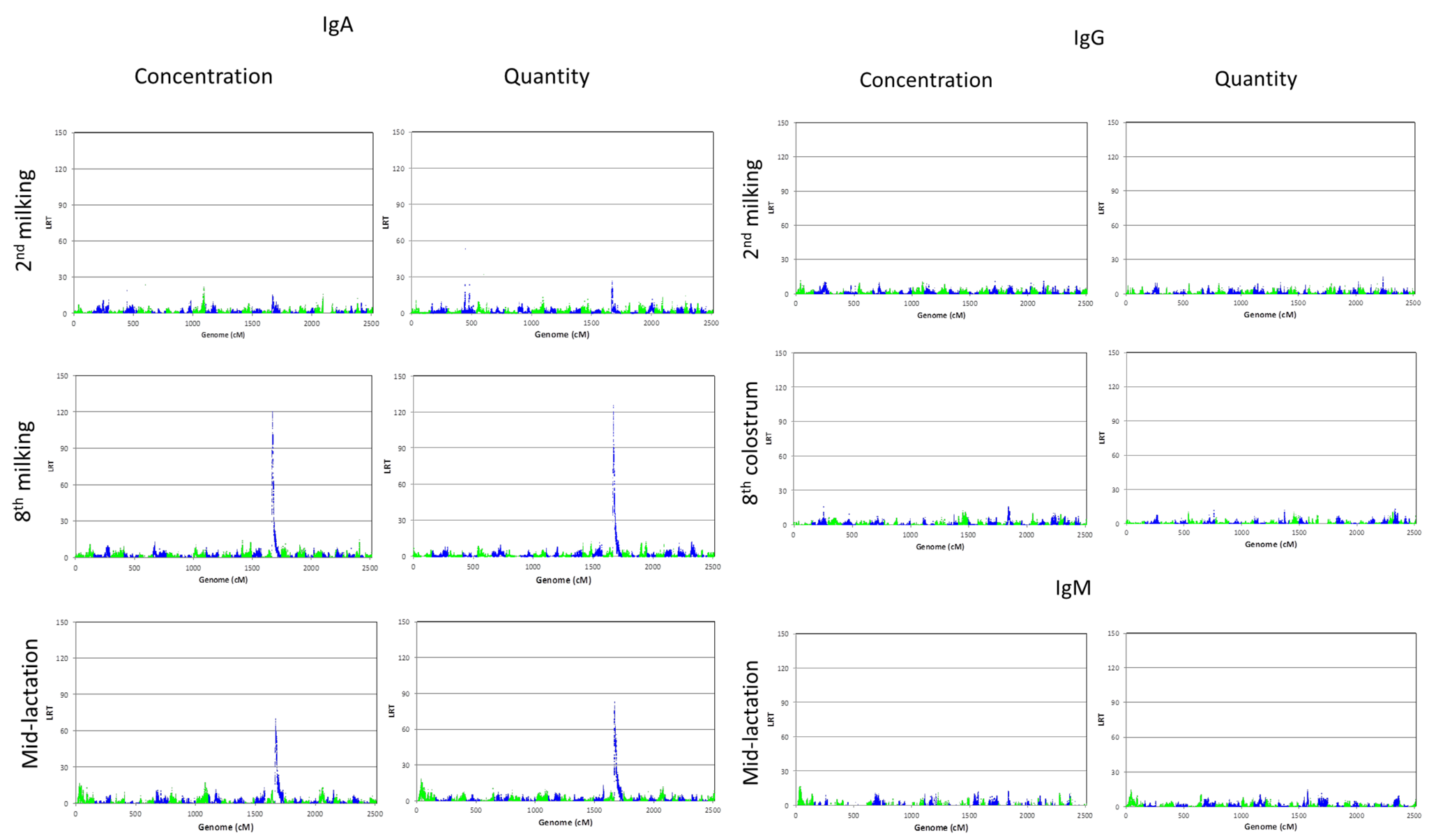

Supplement: Figure S1 — Genome-wide scan for QTL affecting IgA, IgG and IgM. Manhattan plots obtained using a haplotype-based mixed model that simultaneously extracts linkage and LD information and corrects for stratification. For antibody (IgA, IgG or IgM as indicated) concentration (left column) and yield (right column) in 2nd colostrum, 8th colostrum or mid-lactation milk. The LRT threshold for genome-wide significance is 24, while the genome-wide suggestive threshold is 20. (TIF) [file pone.0057219.s001.tif]
